# A CTAB-mediated antisolvent vapor route to shale-like Cs_4_PbBr_6_ microplates showing an eminent photoluminescence†

**DOI:** 10.1039/c9ra10987k

**Published:** 2020-03-09

**Authors:** Yunwei Wei, Wei Zheng, Malik Zeeshan Shahid, Zhixiang Jiang, Yuehua Li, Zhongyao Duan, Guangning Liu, Xun Hu, Cuncheng Li

**Affiliations:** Collaborative Innovation Center for Green Chemical Manufacturing and Accurate Detection, Key Laboratory of Interfacial Reaction & Sensing Analysis in University of Shandong, School of Chemistry and Chemical Engineering, University of Jinan Jinan 250022 Shandong P. R. China chm_licc@ujn.edu.cn; School of Materials Science and Engineering, University of Jinan Jinan 250022 Shandong P. R. China

## Abstract

Compared with nanoscale quantum dots (QDs), the large-sized perovskite crystals not only possess better stability but also are convenient for application exploration. Herein, we develop a facile and efficient antisolvent vapor-assisted recrystallization approach for the synthesis of large-sized Cs_4_PbBr_6_ perovskite crystal microplates. In this method, for the first time, the shale-like Cs_4_PbBr_6_ microplates with lateral dimensions of hundreds of microns are fabricated by employing cetyltriethylammnonium bromide (CTAB) as a morphology-directing agent. FESEM, TEM, and AFM characterizations indicate that the as-obtained shale-like Cs_4_PbBr_6_ microplates are actually formed by 6–8 nm thick Cs_4_PbBr_6_ nanosheets with orientational stacking. Importantly, such highly crystalline Cs_4_PbBr_6_ microplates with shale-like morphology exhibit a narrow and intense green PL emission with a 59% PL quantum yield. Moreover, the planar structure of shale-like Cs_4_PbBr_6_ microplates makes it easy to form a preferred orientation on a substrate, which endow them with promising potential in optoelectronic devices such as lighting and displays.

## Abstract 2

Highly luminescent shale-like Cs_4_PbBr_6_ microplates with hundreds of microns in lateral dimension and formed by thin nanosheets with orientational stacking.

## Graphical abstract

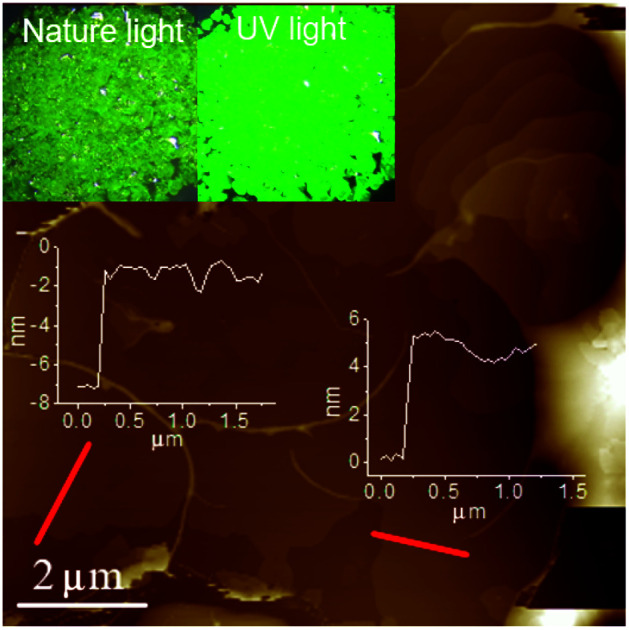

## 1. Introduction

All-inorganic lead halide perovskites with superior photoluminescence (PL) performance are promising functional materials for next-generation optoelectronic devices such as low-cost visible LEDs.^1–4^ Synthesis of perovskite-related materials with high PL quantum yield (PLQY), chromatogram purity, low-threshold lasing, and narrow emission line-width have aroused a wide range of interest in the past few years.^5–9^ Up to now, CsPbX_3_ (X = Cl, Br, I) perovskite quantum dots (QDs), nanocrystals (NCs), nanowires, and nanoplates as well as Cs_4_PbBr_6_ and CsPb_2_Br_5_ perovskite-related materials (PRMs) have been routinely synthesized by various solution processing approaches.^10–17^ Compared with nanoscale materials, the large-sized perovskites not only possess better stability but also are convenient for application exploration.^18–28^ Furthermore, it is well known that the large-sized crystals are very important to identify the essential properties of a material.^18–20^ As a result, more and more researchers have recently begun to focus on the synthesis of microscale or even centimeter-scale perovskite crystals.^18–32^

Currently, pure-phase CsPbBr_3_ perovskite crystals with dimensions of several millimeters were successfully prepared by an antisolvent crystallization method.^19^ Unfortunately, such large sized CsPbBr_3_ crystals are almost non-luminescent.^12^ While, Tang *et al.* reported the perovskite-related CsPb_2_Br_5_ microplates with superior crystallinity, enhanced stability, and tunable optical properties.^29^ It has been revealed that such plate-like perovskites crystals exhibit excellent optical properties due to the absence of tunnel barrier or grain boundary in the planar dimensions.^30^ More recently, as another number of Cs–Pb–Br family, Cs_4_PbBr_6_ PRMs have also attracted enormous attention.^12–15^ It has been revealed that Cs_4_PbBr_6_ PRMs have a higher exciton binding energy (*E*_g_ ≥ 180 meV) which benefits for the recombination of photogenerated charge carriers, resulting in the high efficiency photon emission.^12,33,34^ Interestingly, Cs_4_PbBr_6_ PRMs endows a very high quantum confinement even in the bulk form which make its prospect entirely depends on its crystal structure rather than shape and size.^12,15^ These merits are critical for the high-performance photoelectric devices such as LEDs. Apart from their excellently emissive properties, Cs_4_PbBr_6_ PRMs possess superior high-order nonlinear optical properties in a broad spectral region from 500 to 1500 nm due to the strong confinement effects with a high dipole moment.^35^ Such figures make them showing great potential for multi-photon based imaging as well as optoelectronic devices. Accordingly, the synthesis of Cs_4_PbBr_6_ PRMs received great interest in recent years.

Since the pure-phase Cs_4_PbBr_6_ perovskite solids with green emission were reported by Bakr *et al. via* a low-temperature solution-processed synthesis, many efforts have been devoted to synthesizing luminescent Cs_4_PbBr_6_ perovskite materials.^12–15^ So far, in addition to some micron scale perovskite solid powders with irregular shape,^12–15^ large-sized Cs_4_PbBr_6_ single crystals were also successfully prepared by various solution methods.^18,19,31,32^ For example, Bastiani *et al.* harvested 500 μm pure-phase Cs_4_PbBr_6_ single crystals with smooth surfaces and well-defined rhomboid shape through an antisolvent crystallization process.^31^ An HBr-assisted slow cooling method was developed by Chen *et al.* for the growth of centimeter-sized Cs_4_PbBr_6_ crystals with embedded highly luminescent CsPbBr_3_ NCs.^18^ As is known, unlike CsPbBr_3_ possessing a higher symmetry simple cubic structure with connected corner-sharing PbBr_6_^4−^ octahedra, the PbBr_6_^4−^ octahedra in Cs_4_PbBr_6_ are fully isolated from each other in the crystal lattice with interspersed Cs^+^ cations.^12,13,15,33,34^ Therefore, the large-sized Cs_4_PbBr_6_ PRMs reported generally have an irregular or rhomboid shape but the plate-like Cs_4_PbBr_6_ perovskite crystals are rarely reported until now.^12–15,18,19,31,32^ Therefore, developing an effective route for the synthesis of large scale plate-like Cs_4_PbBr_6_ perovskite materials with highly photoluminescence is of great significance for practical applications.^17,21–25,29,30^

Herein, for the first time, we report a facile and efficient route for the synthesis of highly luminescent shale-like Cs_4_PbBr_6_ microplates with hundreds of microns in lateral dimension and tens of microns in thickness. The synthesis of shale-like Cs_4_PbBr_6_ microplates was designed according to the supersaturated recrystallization method with the assistance of antisolvent vapor, in which, the heated toluene vapor is diffused into the DMF solution containing PbBr_2_, CsBr, and CTAB. Although the overall thickness of the as-synthesized shale-like Cs_4_PbBr_6_ microplates is dozens of microns, they are actually formed by 6–8 nm thick Cs_4_PbBr_6_ nanosheets with orientational stacking. Such shale-like Cs_4_PbBr_6_ single crystalline microplates not only have superior optical properties, but easy to form a preferred orientation on a substrate, which endows them with promising potential in optoelectronic devices such as lighting and displays.

## 2. Experimental section

### 2.1 Materials and chemicals

Lead bromide (PbBr_2_, Macklin, 99%), cesium bromide (CsBr, Macklin, 99%), cetyltrimethylammonium bromide (CTAB, Macklin, 99%), *N*,*N*-dimethylformamide (DMF, Aladdin, 99.5%), toluene (Sinopharm Chemical Reagent Co. Ltd., 99.5%), commercial red phosphors (Xiamen Chen Zhou Optoelectronics Technology Co. Ltd., China), silicone gel A and B (Shenzhen looking long technology Co. Ltd., China), blue GaN chips (Shenzhen looking long technology Co. Ltd., China) were used as received without further purification.

### 2.2 Synthesis of shale-like Cs_4_PbBr_6_ perovskite microplates

The synthetic procedure for shale-like Cs_4_PbBr_6_ perovskite microplates was as follow: the precursor solution was firstly prepared by dissolving a given amount of PbBr_2_, CsBr, and CTAB into DMF under the sonication. The final concentrations of PbBr_2_, CsBr, and CTAB in the precursor were 10 mM, 10 mM, and 15 mM, respectively. Subsequently, 2 mL of the as-prepared precursor solution and 5 mL toluene as antisolvent were separately introduced to one of glass vessels in a connector as illustrated in Fig. S1.† After sealing both of vessels, the vessel containing toluene was heated at 80 °C in an oil-bath, while the vessel with precursor was put into a 25 °C water-bath. In this case, the toluene vapor was gradually evaporated into the precursor solution during the reaction process. After about 36 h, the shale-like microplates with yellow-green color were yielded at the bottom of the vessel in the water-bath. Finally, the yellow-green microplates were harvested and washed repeatedly with toluene for characterization and performance evaluation. Further experiments were performed to reveal the roles of CTAB on the synthesis of shale-like Cs_4_PbBr_6_ perovskite microplates.

### 2.3 Characterizations and measurements

To determine the crystal structure of the as-prepared perovskite crystals, X-ray diffraction (XRD) patterns were recorded on a Rigaku Ultima IV X-ray diffractometer with Cu Kα radiation (*λ* = 1.5418 Å) by painting the as-prepared products on a silicon wafer. The morphologies of the products were characterized by transmission electron microscopy (TEM, JEOL JEM-1400) and high-resolution transmission electron microscopy (HRTEM, JEOL JEM-2100). The samples for TEM and HRTEM observations were prepared by putting the products on a thin carbon film coated copper grids. Field-emission scanning electron microscope (FESEM) and energy-dispersive X-ray (EDX) elemental mapping were carried out on ZEISS Gemini-300. Atomic force microscope (AFM, Bruker Multimode-8) was carried out to obtain the thickness of the as-prepared shale-like Cs_4_PbBr_6_ microplates. The absorption spectrum was recorded on Shimadzu UV-3101PC with an integrated sphere by using diffuse-reflectance mode. The steady-state PL was measured using a Shimadzu RF-6000 spectrofluorometer. The PLQY and PL lifetime of the as-prepared samples were characterized by using an Edinburgh FLS920 multifunction steady state and transient state fluorescence spectrometer. The excitation wavelength used for all the PL measurements were set at 360 nm. Optical microscope and fluorescent pictures of shale-like Cs_4_PbBr_6_ perovskite microplates were recorded on a Nikon ECLIPSE-E100 with an UV lamp. The photoelectric parameters including correlated color temperature (CCT) and CIE coordinates of the fabricated devices were evaluated on a spectroradiometer system (EVERFINE, SPIC-200).

### 2.4 Fabrication of white light-emitting diode (WLED) device

To fabricate WLED device, the silicone gel A and B were firstly blended with an A to B volumetric ratio of 1 : 4. The as-prepared shale-like Cs_4_PbBr_6_ perovskite microplates and the commercial red phosphors were introduced into the above silicone mixture gel to form uniform colloids. A given amount of the as-prepared green-red emitting colloids were finally painted onto a blue GaN chip and then dried in a vacuum oven at 60 °C.

## 3. Results and discussion

### 3.1 Morphological characterization of shale-like Cs_4_PbBr_6_ microplates

In this work, a CTAB-mediated antisolvent vapor approach was designed for the synthesis of shale-like Cs_4_PbBr_6_ perovskite microplates by using toluene as antisolvent and DMF as precursor solvent. As presented in Fig. S1,† the precursor solution and toluene are separately added to one of glass vessels in a connector. To enable toluene diffusion into the precursor solution, the toluene was heated at 80 °C and the precursor solution was kept at 25 °C. As the reaction time increasing, the volume of liquid in toluene container decreased gradually while the volume of the liquid in the precursor solution container increased, which indicated that toluene was transferred into the precursor. Interestingly, the yellow-green crystals were precipitated from the precursor after reaction about 36 h and the upper solution appears clear and colorless.

**Fig. 1:**
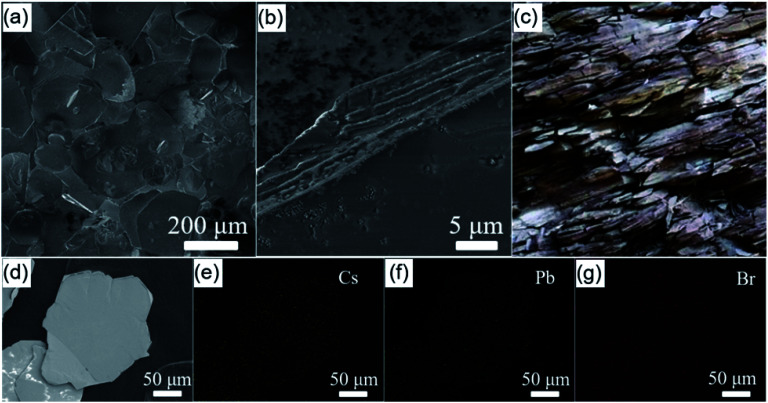
(a) Low- and (b) high-magnification FESEM images of the obtained shale-like Cs_4_PbBr_6_ perovskite microplates. (c) Photograph of natural shales in the Wufeng mountain in Jinan. (d) FESEM image of a random Cs_4_PbBr_6_ microplate and the corresponding EDS mapping images for (e) Cs element, (f) Pb element, (g) Br element. Scale bars for (a), (b), and (d)–(g) are 200 μm, 5 μm, 50 μm, respectively.

FESEM observation was firstly employed to get the morphology of the as-obtained crystals. As presented in Fig. 1a and b, FESEM images show that almost all the products are plate-like particles with hundreds of microns in dimension and tens of microns in thickness. The lateral FESEM image presented in Fig. 1b reveals that the as-obtained microplates have a multilayer structure. Specifically, a single microplate composes of countless oriented stacking ultrathin nanosheets, which is similar to the natural shale demonstrated in Fig. 1c. The EDS elemental mapping images for a random microplate indicates that the Cs, Pb, and Br elements are homogeneously distributed in the whole plates (Fig. 1d–g), reflecting that the as-synthesized microplates is a kind of cesium lead bromide compounds. The weight percentages of Cs, Pb, and Br elements in the product are 42.8%, 19.0%, 38.2%, respectively, corresponding to a Cs : Pb : Br atomic ratio of 4 : 1.1 : 5.9 (Fig. S2†), which is close to the stoichiometric ratio of Cs, Pb, and Br elements in Cs_4_PbBr_6_.

**Fig. 2:**
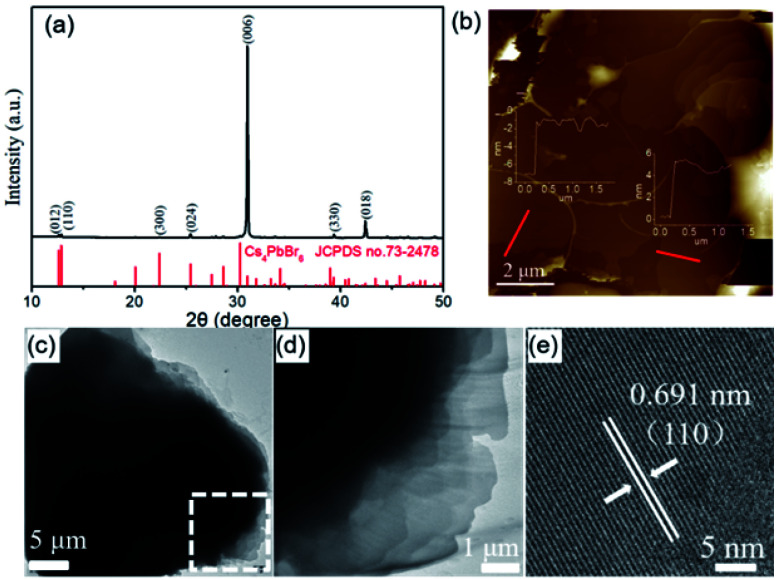
(a) XRD pattern of the as-obtained product. (b) AFM and (c) TEM images of a single shale-like Cs_4_PbBr_6_ perovskite microplate, and (d) local high magnification TEM image, (e) HRTEM image in (c). Inset in (b) shows the corresponding thickness of the monolayer sheets in the shale-like Cs_4_PbBr_6_ microplates. Scale bars for (b), (c), (d), and (e) are 2 μm, 5 μm, 1 μm, and 5 nm, respectively.

The XRD measurement was carried out to reveal the crystallographic structure of the as-obtained cesium lead bromide compound. As shown in Fig. 2a, all the XRD diffraction peaks are well indexed to rhombohedral phase Cs_4_PbBr_6_ (JCPDS no. 73-2478). Interestingly, the (006) diffraction peak of Cs_4_PbBr_6_ is extremely higher than other peaks. The peak intense is obviously different from that in the JCPDS. Such variation is indeed frequently observed in the XRD diffraction patterns of plate-like materials such as Au and BiOCl nanosheets.^36,37^ This is because that, unlike the quasi-spherical particles, the particles with plate-like morphology prefer to form a preferential orientation when they are dispersed on a slide. This thus results in the lattice planes which parallel to the surface of the substrate have an intense XRD diffraction peak. On the basis of the above results and analyses, we infer that the obtained Cs_4_PbBr_6_ microplates are abundant with {006} planes but they have a preferred orientation with 〈006〉 direction on the substrate due to their planar geometry shape. Furthermore, it can be rationally inferred that the top and bottom surfaces of Cs_4_PbBr_6_ microplates paralleled to the substrate are {006} lattice planes.

The morphological features of Cs_4_PbBr_6_ microplates were further characterized by TEM and AFM. The TEM and AFM images also illustrate the as-obtained Cs_4_PbBr_6_ is a multilayer microplates constructed by ultrathin nanosheets (Fig. 2b–d). The thickness of the ultrathin nanosheets is measured by AFM at the edge of a random microplate (Fig. 2b). The AFM images and line profiles indicate that the thickness of the ultrathin nanosheets, the elementary units for Cs_4_PbBr_6_ microplates, is about 6–8 nm. Moreover, HRTEM image shown in Fig. 2e verifies that the interplanar distances was about 0.691 nm, which can be indexed as the {110} planes of the rhombohedral phase Cs_4_PbBr_6_. According to the above morphology characterizations, the product obtained by the CTAB-mediated antisolvent vapor approach presented here is thus denoted as shale-like Cs_4_PbBr_6_ microplates.

### 3.2 The optical properties of shale-like Cs_4_PbBr_6_ microplates

**Fig. 3:**
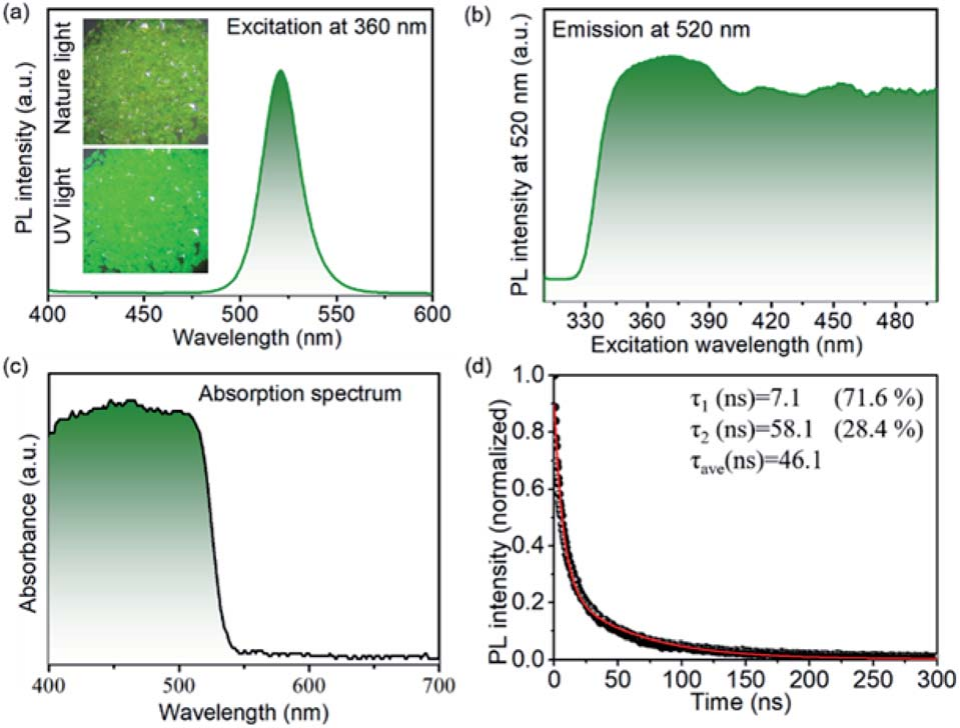
(a) PL emission spectrum, (b) excitation spectrum, (c) absorption spectrum, and (d) time-resolved PL decay of shale-like Cs_4_PbBr_6_ perovskite microplates. Inset in Fig. 1a shows the optical microscope picture under nature light (up) and the fluorescent picture under 365 nm UV light (down) of Cs_4_PbBr_6_ perovskite microplates.

The inset in Fig. 3a presents the optical microscope picture of the shale-like Cs_4_PbBr_6_ microplates under nature light and fluorescent picture under 365 nm UV light. It is clearly observed that shale-like Cs_4_PbBr_6_ microplates appears to be yellow-green under the natural light and exhibits a bright green emission under 365 nm UV light, indicating that the products possess an excellent photoluminescence.^15^ The optical properties of shale-like Cs_4_PbBr_6_ microplates were further studied by photoluminescence spectrum and UV-Vis absorption. The as-prepared shale-like Cs_4_PbBr_6_ microplates exhibit a single, intense, and stable emission at 520 nm when the excitation wavelengths is greater than 350 nm (Fig. 3a and b). In addition, the absorption spectrum in Fig. 3c with an absorption edge onset at 540 nm is observed, which was consistent with the literature reports.^12^ Furthermore, the PLQY of the shale-like Cs_4_PbBr_6_ microplates is about 59% obtained by a spectrofluorometer equipped with an integrated sphere under the excitation of 360 nm illumination (Fig. S3†). Fig. 3d shows the time-resolved decay curve of the as-obtained shale-like Cs_4_PbBr_6_ microplates, which is well fitted by a biexponential function:
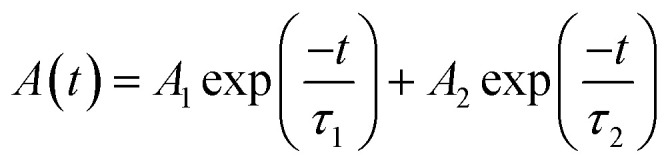
where *A*(*t*) is the PL intensity at time *t*, *A*_1_, and *A*_2_ are constants corresponding to the fractions of slow decay and long decay, respectively.^12,15,38,39^ It is clearly observed that shale-like Cs_4_PbBr_6_ microplates exhibit a short PL lifetime (*τ*_1_) of 7.1 ns with a percentage of 71.6% and a long PL lifetime (*τ*_2_) of 58.1 ns with a percentage of 28.4%. Moreover, the average PL lifetime of shale-like Cs_4_PbBr_6_ microplates is calculated to be 46.1 ns.

### 3.3 Roles of CTAB for the formation of shale-like Cs_4_PbBr_6_ microplates

**Fig. 4:**
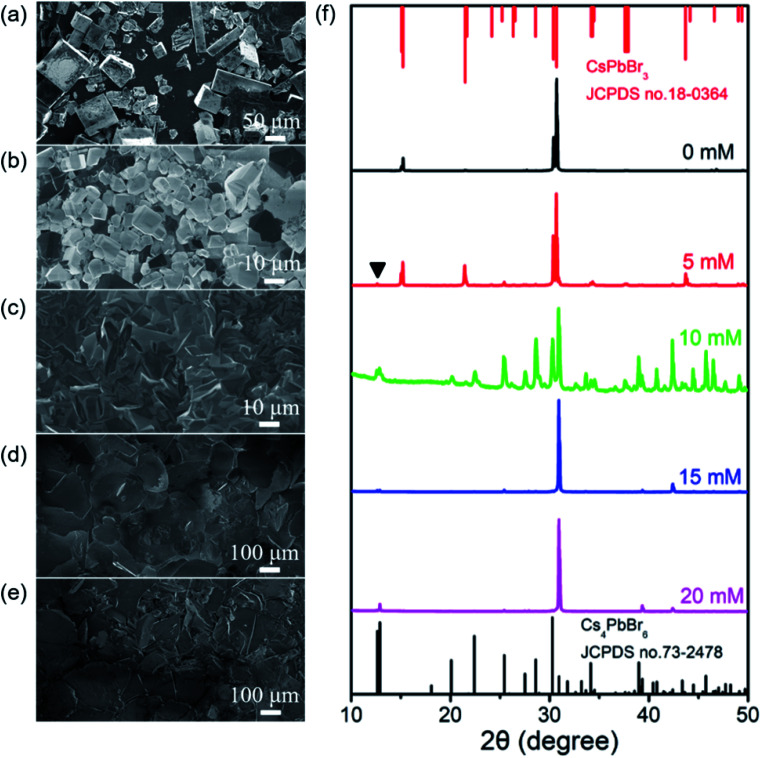
(a)–(e) SEM images and (f) XRD patterns of the perovskite crystals obtained with different concentration of CTAB from 0 mM to 20 mM. Stick patterns in Fig. 3g: standard XRD of CsPbBr_3_ (JCPDS no. 18-0364, red lines) and Cs_4_PbBr_6_ (JCPDS no. 73-2478, black lines). Scale bars for (a), (b), (c), (d), and (e) are 50 μm, 10 μm, 10 μm, 100 μm, and 100 μm respectively.

Further experiments revealed that CTAB play a key role for the synthesis of the shale-like Cs_4_PbBr_6_ microplates. Fig. S4a and S4b† present the results of the product obtained by using a precursor solution containing PbBr_2_ and CsBr but without using CTAB. In this case, the final color of the product appeared brown and almost no luminescence was observed under UV light. The corresponding FESEM image and XRD pattern verify that the final product yielded in the absence of CTAB is rectangular CsPbBr_3_ microcrystals with edge lengths range from tens to hundreds of microns, as typically illustrated in Fig. 4a and f. Almost no luminescent signal for such big CsPbBr_3_ crystals is detected in PL measurement (Fig. S4k†), agreeing well with the fluorescent picture (Fig. S4a†) and literatures.^12,15,19^ This result unambiguously reveals that CTAB is very crucial for the formation of shale-like Cs_4_PbBr_6_ microplates with highly photoluminescence.

To further explore the role of CTAB in the formation of shale-like Cs_4_PbBr_6_ microplates, a series of experiments were performed by varying its concentration in the initial precursor and keeping other parameters invariant. When CTAB concentration is 5 mM, besides a small amount of plate-like crystals with green emission, the product is still predominately made up of non-luminescent CsPbBr_3_ microcrystals but its grain size becomes smaller (Fig. 4b, S4c, and S4d†). Interestingly, a weak PL emission at 520 nm is detected in the corresponding PL spectrum in the present case (Fig. S4k†). The PL emission of the harvested products is further enhanced as the increase of CTAB concentration and reaches the maximum if the CTAB concentration is above 15 mM. This is well consistent with the observation of the samples under nature and UV light. A distinct color evolution from orange to yellow-green under nature light (Fig. S4c, S4e, S4g, and S4i†) and the enhancement of green emission under UV light (Fig. S4d, S4f, S4h, and S4j†) are clearly observed as the CTAB concentration is increased from 5 mM to 20 mM. Correspondingly, XRD measurements indicate that the crystalline phases of the as-obtained products are changed from orthorhombic CsPbBr_3_ to rhombohedral Cs_4_PbBr_6_ compound (Fig. 4f). Moreover, the morphology of the products evolution from small cube (Fig. 4b) to small plate-like crystals (Fig. 4b), and final obtained large-sized microplates (Fig. 4d and e). Furthermore, the final products obtained by high concentration CTAB are dominated by single-crystalline Cs_4_PbBr_6_ compound with {006} lattice planes as the basal surfaces. FESEM observations indicate that the population of the shale-like Cs_4_PbBr_6_ microplates is increased as the increase of CTAB concentration (Fig. 4a–e). This further confirms that the generation of the shale-like Cs_4_PbBr_6_ microplates does closely relate to CTAB.

As one of the most common shape-directing agents, the cationic surfactant CTAB has always been a widely utilized in the synthesis of functional materials.^40,41^ Fig. S5a and S5b† show the results of the same amount of CTAB were respectively added in DMF and toluene. We find that the CTAB in DMF solution is clear and colorless, whereas the CTAB in toluene appears turbid with white color. This suggests that CTAB can dissolve into DMF easily but do not dissolve into toluene. Similarly, the solubility of CsBr and PbBr_2_ in DMF and toluene is also significantly different: DMF is a good solvent for CsBr and PbBr_2_ while toluene is a very poor solvent for them.^10,13^ As aforementioned, in the present synthesis, toluene can be easily entered into the DMF precursor solution containing CsBr, PbBr_2_, and CTAB through an evaporation process. Interestingly, the result illustrated in Fig. S5c† indicates that CTAB can be recrystallized to form white micelles if toluene is introduced into DMF solution containing CTAB. Moreover, the recrystallization of Cs^+^, Pb^2+^, Br^−^ ions from DMF solution by using toluene as an anti-solvent has also been well evidenced in recent researches.^10,13,15^ Obviously, with the reaction time increasing, the precursor ions (*e.g.* Cs^+^, Pb^2+^, Br^−^) and CTAB will be recrystallized together due to the increase of toluene content in the reaction solution. Furthermore, it is well known that (i) CTAB can be facilely self-assembled to form layered micelles when its concentration in a solution is higher than the critical concentration^40,41^ and (ii) the Gibbs free energy of heterogeneous nucleation is generally lower than that of homogeneous nucleation.^42,43^ On the basis of the above information and our experimental results, we infer that, under a slow crystallization process like our case, the layered structure CTAB micelles may act as the soft templates and in turn direct the growth process of cesium lead bromide compound, thus leading to the formation of shale-like Cs_4_PbBr_6_ microplates. Specially, shale-like Cs_4_PbBr_6_ microplates are exclusively produced when the CTAB concentration in the initial precursor is greater than 15 mM (Fig. 4). According to our results and the above analysis, the morphologies of Cs_4_PbBr_6_ PRMs formed with various CTAB concentrations are summarized and schematically illustrated in the ESI (Fig. S6†).

### 3.4 Application in WLED devices

**Fig. 5:**
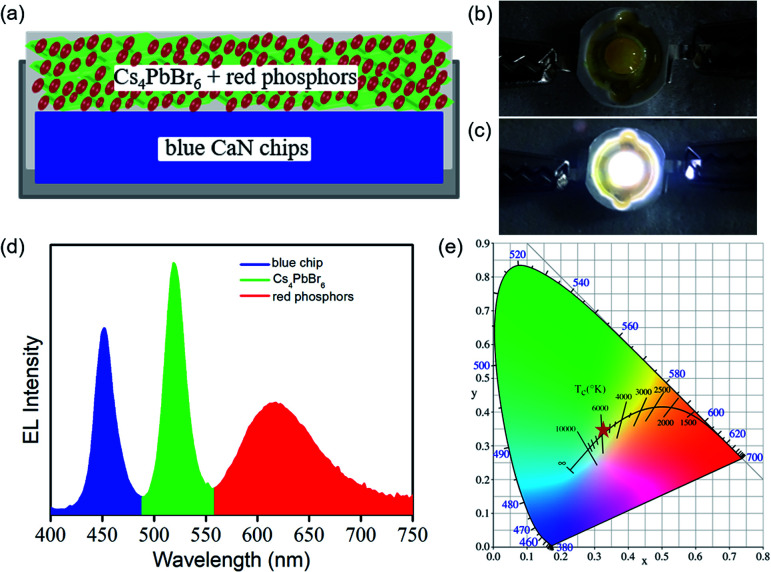
(a) Schematic diagram of the configuration of WLEDs device designed by combining a blue GaN chip with shale-like Cs_4_PbBr_6_ microplates and red phosphors color converter. (b) The photograph of the as-fabricated WLED device and (c) the WLED driven by 2.5 V operation voltage with an operation current of 2 mA. (d) The corresponding EL spectrum of the as-fabricated WLED device. (e) CIE coordinates of the WLED devices in CIE 1931 space.

To illustrate their potential applications, a prototype white light-emitting diode (WLED) device were fabricated by using a 450 nm emissive GaN chip as blue light source. Fig. 5a presents the framework of WLED device. Fig. 5b and c present the photograph for the prototype of WLED device in non-working and working state. It is clearly observed that the shale-like Cs_4_PbBr_6_ microplates-based WLED device exhibits a saturated white emission at an operation current of 2 mA (Fig. 5c). Fig. 5d shows the corresponding electroluminescence (EL) spectrum of the WLED device under working state. On the basis of the WLED device framework presented in Fig. 5a, we attribute the luminescent peaks at 450 nm, 520 nm, and 615 nm in the EL spectrum (Fig. 5d), respectively to the emission from GaN chip, Cs_4_PbBr_6_ perovskite, and commercial red phosphors. Moreover, as demonstrated in Fig. 5e, the as-fabricated WLED device exhibits a cold white light with a color temperature of 5612 K, and the CIE 1931 color coordinate of (0.33, 0.34), which is close to the standard white color (0.33, 0.33).^15,18,44^ Such results described above suggest that the great potential applications of shale-like Cs_4_PbBr_6_ microplates in lighting and display devices.

## 4. Conclusions

In summary, in this work, we develop a facile and efficient antisolvent vapor-assisted crystallization approach for the synthesis of shale-like Cs_4_PbBr_6_ microplates with large size. Interestingly, the shale-like Cs_4_PbBr_6_ microplates with highly PL are rationally synthesized by using toluene as anti-solvent and DMF as precursor solvent. Structural characterizations by FESEM, TEM, and AFM indicate that the as-obtained shale-like Cs_4_PbBr_6_ microplates are constructed by 6–8 nm thick Cs_4_PbBr_6_ nanosheets with orientational stacking. The cationic surfactant CTAB as a morphology-directing agent is found to be crucial role for the formation of shale-like Cs_4_PbBr_6_ microplates. Importantly, such shale-like Cs_4_PbBr_6_ single crystalline microplates on a substrate are easy to form a preferred orientation film with 〈006〉 direction due to their planar structure, which makes them easy to process for application exploration. Finally, a prototype WLED device is successfully fabricated by combining the shale-like Cs_4_PbBr_6_ microplates as the green emitters, commercial red phosphors as the red emitters, and blue emitting GaN chips as the blue emitters, which shows their great potential application in optoelectronic devices such as lighting and displays.

## Conflicts of interest

There are no conflicts of interest to declare.

## Supplementary Material

RA-010-C9RA10987K-s001
