# Supplementary material for: A CTAB-mediated antisolvent vapor route to shale-like Cs4PbBr6 microplates showing an eminent photoluminescence
Source: RSC Adv. 2020 Mar 9;10(17):10023–9. doi: 10.1039/c9ra10987k (PMC9050374; doi:10.1039/c9ra10987k)
Supplement: RA-010-C9RA10987K-s001 [file RA-010-C9RA10987K-s001.pdf]

Electronic Supplementary Material (ESI) for RSC Advances.
This journal is © The Royal Society of Chemistry 2020

Supporting Information

A CTAB-Mediated Antisolvent Vapor Route to Shale-Like Cs_4PbBr_6 Microplates Showing an Eminent Photoluminescence

Yunwei Wei,[a] Wei Zheng,[b] Malik Zeeshan Shahid,[a] Zhixiang Jiang,[b] Yuehua Li,[a] Zhongyao Duan,[a] Guangning Liu,[a] Xun Hu,[b] Cuncheng Li*[a]

[a] Collaborative Innovation Center for Green Chemical Manufacturing and Accurate Detection, Key Laboratory of Interfacial Reaction & Sensing Analysis in University of Shandong, School of Chemistry and Chemical Engineering, University of Jinan, Jinan 250022, Shandong, P. R. China

[b] School of Materials Science and Engineering, University of Jinan, Jinan 250022, Shandong, P. R. China

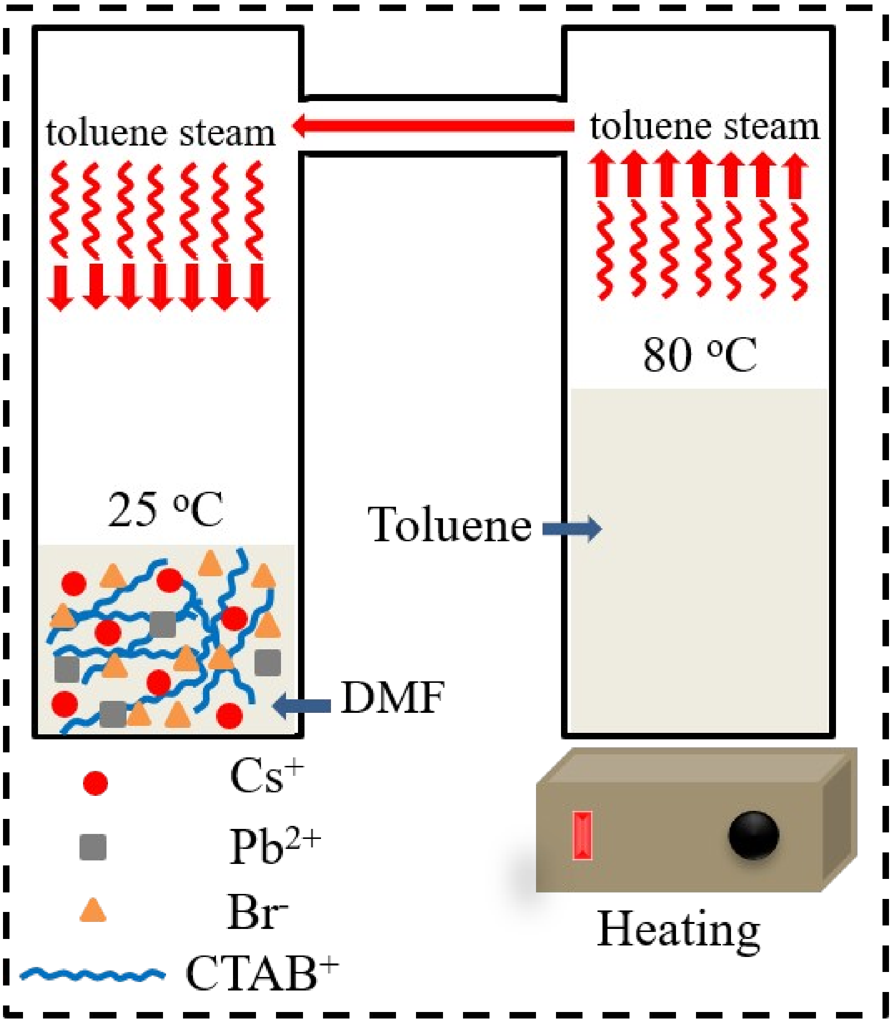

Figure S1. Schematic illustration for the synthesis process of shale-like Cs_4PbBr_6 perovskite microplates.

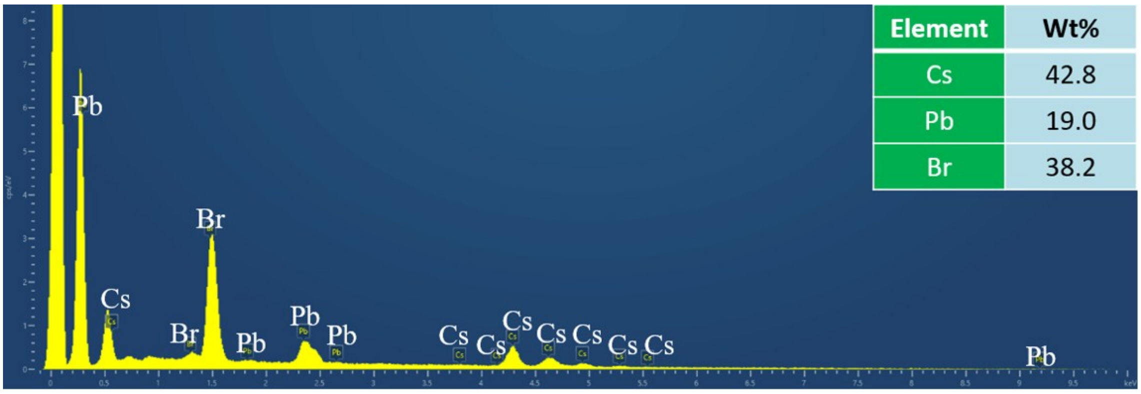

Figure S2. The corresponding EDS spectrum of the shale-like Cs_4PbBr_6 perovskite microplates in Figure 1d.

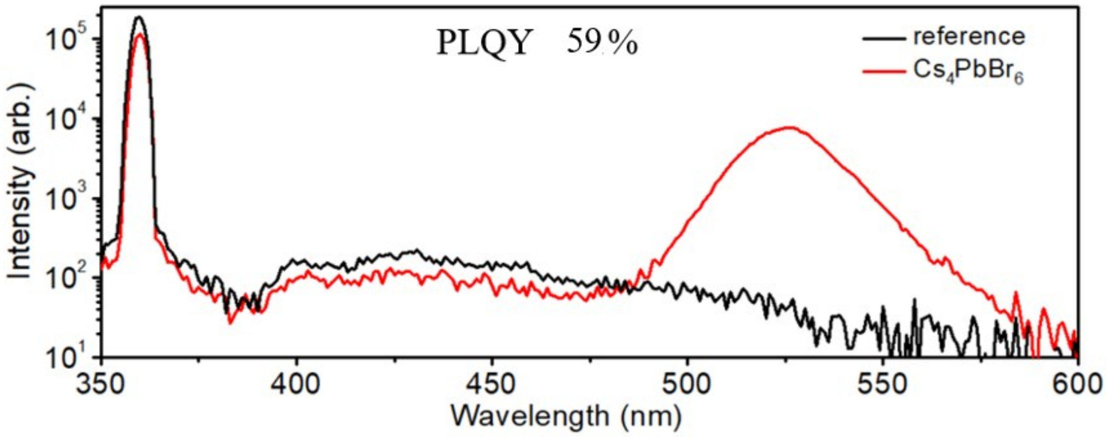

Figure S3. Photoluminescence quantum yield of the as-prepared shale-like Cs_4PbBr_6 perovskite microplates. Black line: reference spectrum, red line: Cs_4PbBr_6 perovskite microplates.

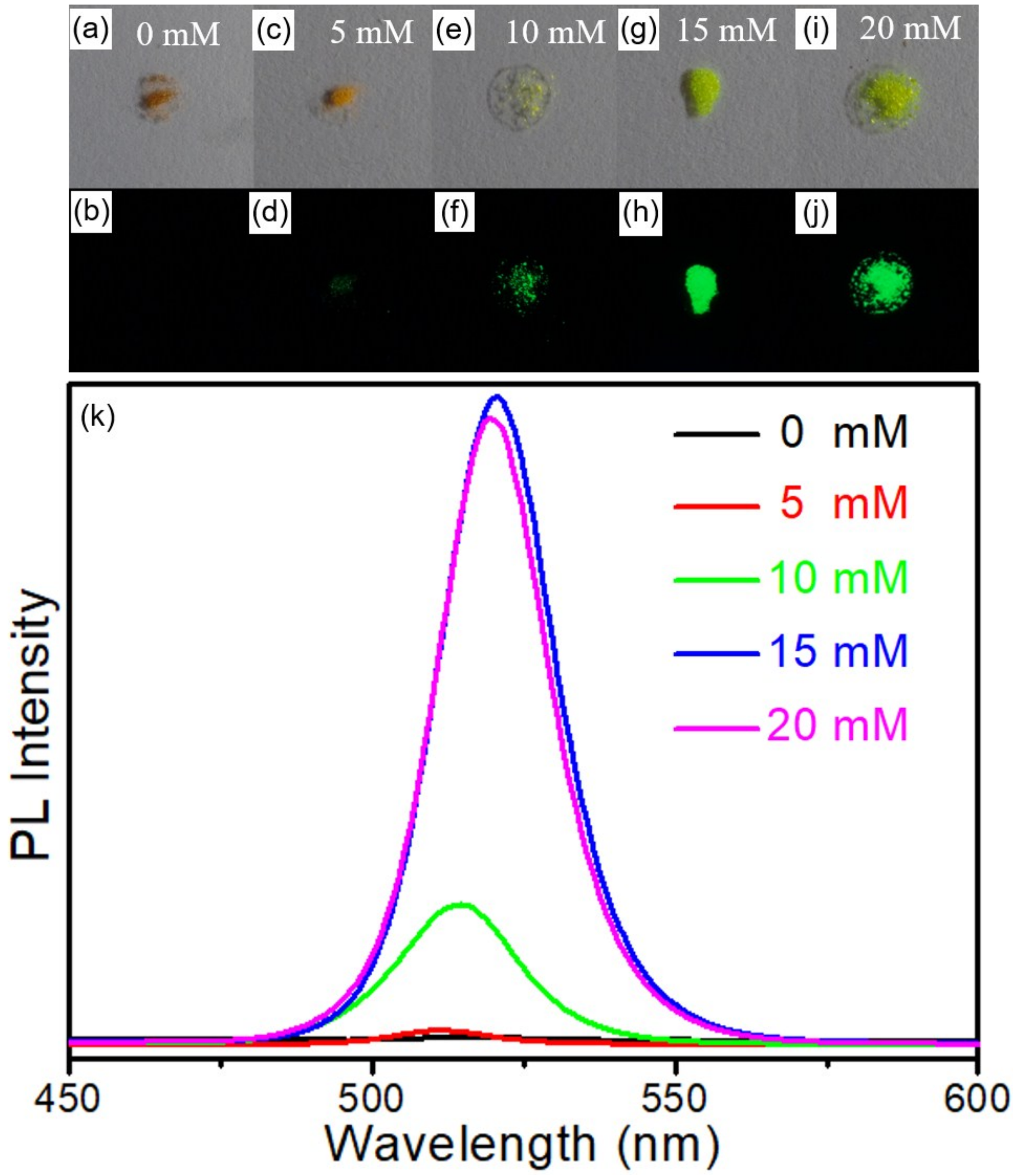

Figure S4. (a), (c), (e), (g), (i) optical microscope pictures under nature light, and (b), (d), (f), (h), (j) fluorescent pictures under 365 nm UV light and (k) PL emission spectra of the perovskite crystals with different concentrations of CTAB from 0 mM to 20 mM.

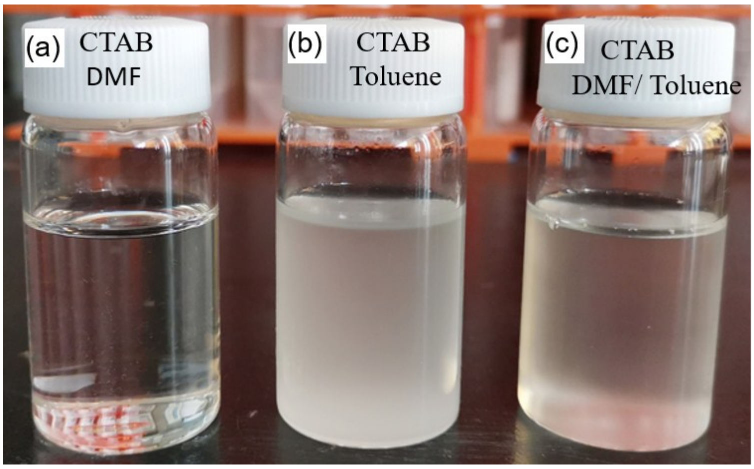

Figure S5. CTAB in (a) DMF, (b) toluene, and (c) the mixed solution of DMF and toluene with volume ratio 2:5.

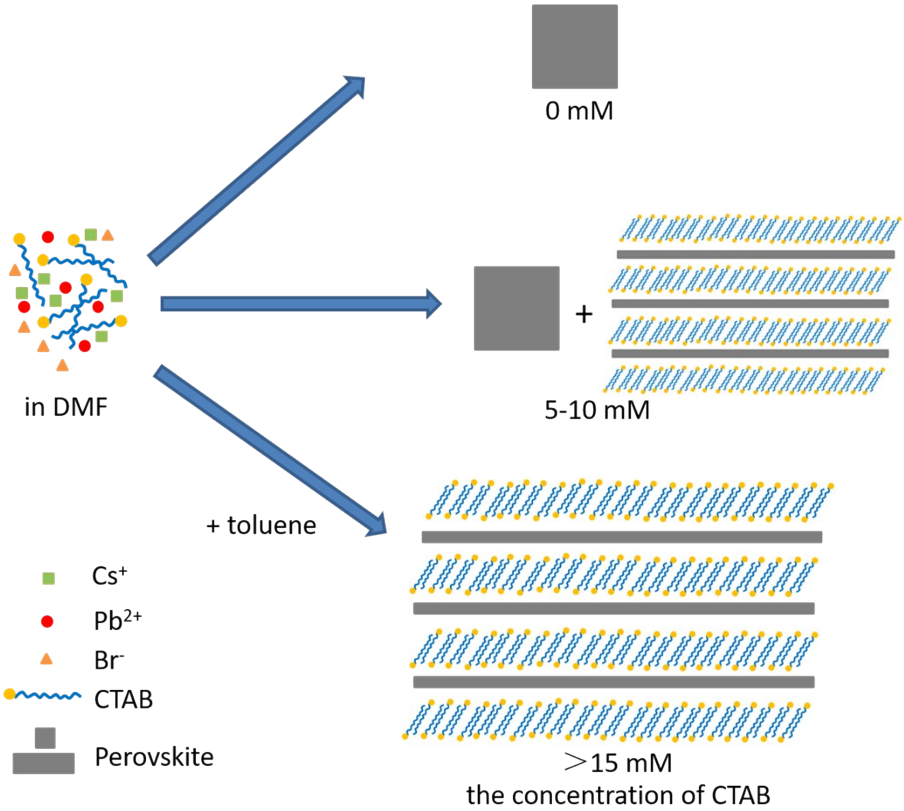

Figure S6. Schematic illustration for the morphologies of Cs_4PbBr_6 perovskite-related materials obtained with CTAB of different concentrations.
